# Neurophysiology of Avian Sleep: Comparing Natural Sleep and Isoflurane Anesthesia

**DOI:** 10.3389/fnins.2019.00262

**Published:** 2019-03-28

**Authors:** Jacqueline van der Meij, Dolores Martinez-Gonzalez, Gabriël J. L. Beckers, Niels C. Rattenborg

**Affiliations:** ^1^Avian Sleep Group, Max Planck Institute for Ornithology, Seewiesen, Germany; ^2^Cognitive Neurobiology and Helmholtz Institute, Utrecht University, Utrecht, Netherlands

**Keywords:** *Columba livia*, avian brain, visual hyperpallium, NREM sleep, isoflurane anesthesia, slow-waves, burst suppression, propagation

## Abstract

Propagating slow-waves in electroencephalogram (EEG) or local field potential (LFP) recordings occur during non-rapid eye-movement (NREM) sleep in both mammals and birds. Moreover, in both, input from the thalamus is thought to contribute to the genesis of NREM sleep slow-waves. Interestingly, the general features of slow-waves are also found under isoflurane anesthesia. However, it is unclear to what extent these slow-waves reflect the same processes as those giving rise to NREM sleep slow-waves. Similar slow-wave spatio-temporal properties during NREM sleep and isoflurane anesthesia would suggest that both types of slow-waves are based on related processes. We used a 32-channel silicon probe connected to a transmitter to make intra-cortical recordings of the visual hyperpallium in naturally sleeping and isoflurane anesthetized pigeons (*Columba livia*) using a within-bird design. Under anesthesia, the amplitude of LFP slow-waves was higher when compared to NREM sleep. Spectral power density across all frequencies (1.5–100 Hz) was also elevated. In addition, slow-wave coherence between electrode sites was higher under anesthesia, indicating higher synchrony when compared to NREM sleep. Nonetheless, the spatial distribution of slow-waves under anesthesia was more comparable to NREM sleep than to wake or REM sleep. Similar to NREM sleep, slow-wave propagation under anesthesia mainly occurred in the thalamic input layers of the hyperpallium, regions which also showed the greatest slow-wave power during both recording conditions. This suggests that the thalamus could be involved in the genesis of slow-waves under both conditions. Taken together, although slow-waves under isoflurane anesthesia are stronger, they share spatio-temporal activity characteristics with slow-waves during NREM sleep.

## Introduction

In mammals, non-rapid eye-movement (NREM) sleep is characterized by the slow alternation in neuronal membrane potentials between hyperpolarized down-states with neuronal quiescence and depolarized up-states with action potentials, which gives rise to slow-waves (0.5–4.5 Hz) in local field potential (LFP) and electroencephalogram (EEG) recordings (Steriade et al., 1993c) that propagate horizontally across the neocortex (Massimini et al., 2004; Murphy et al., 2009; Nir et al., 2011). Our understanding of the neurophysiology of slow-waves is mostly based on EEG, LFP, and intracellular recordings performed under anesthesia. Previous experiments on rats and cats anesthetized with urethane or ketamine–xylazine revealed slow cortical activity similar to that found during NREM sleep (Steriade et al., 1993a,b,c; Contreras and Steriade, 1995; Haider et al., 2006, 2007; Volgushev et al., 2006; Hasenstaub et al., 2007; Luczak et al., 2007; Chauvette et al., 2010, 2011; Sharma et al., 2010). Under higher doses of anesthesia, this slow cortical activity occurs in bursts that alternate with low-amplitude periods of suppressed activity (i.e., burst suppression; Swank and Watson, 1949; Amzica, 2015). Although slow-waves during NREM sleep and anesthesia appear visually similar, a quantitative comparison in cats revealed multiple distinct features between the two brain states (Chauvette et al., 2011). Anesthesia (urethane and/or ketamine-xylazine) induced slow-waves were more rhythmic (i.e., intervals between slow-waves became more regular) and occurred more synchronously across cortical areas than during NREM sleep (Steriade et al., 1993c; Wolansky et al., 2006; Clement et al., 2008; Chauvette et al., 2011). Overall slow-wave amplitude was higher under anesthesia and area specific differences in slow-wave amplitude observed during NREM sleep were reduced under anesthesia (Chauvette et al., 2011). In addition, while power in the slow/delta (0.1–4 Hz) and spindle (8–14 Hz) frequency ranges decreased, the power in the gamma band (30–100 Hz) increased. Finally, anesthesia increased the duration of the hyperpolarization periods of slow-waves when compared to NREM sleep (Chauvette et al., 2011).

In addition to investigating the spectral differences occurring under anesthesia, several studies have also examined the layer-specific aspects and propagation of slow-waves under anesthesia and NREM sleep, although a direct comparison between conditions is lacking. In both anesthetized and naturally sleeping cats, for instance, similar intra-columnar propagation patterns are found with slow-wave up-states that appear first within layer 5 (Chauvette et al., 2010), a layer that together with layer 4 receives thalamic input (Constantinople and Bruno, 2013), and then propagate vertically within a column to layer 4 and the supragranular layers (Chauvette et al., 2010; Constantinople and Bruno, 2013; Capone et al., 2017; Fiáth et al., 2018). Contrary to the intra-columnar propagation of up-states examined during NREM sleep and anesthesia, the layer-specific horizontal propagation of up-states has only been examined *in vivo* in anesthetized rodents (Luczak et al., 2007; Sakata and Harris, 2009; Reyes-Puerta et al., 2015).

At present, our understanding of the network properties underlying slow-waves and their propagation under NREM sleep and anesthesia is limited to the few mammalian studies described above. Interestingly, birds exhibit mammalian-like, homeostatically regulated EEG slow-waves during NREM sleep (Lesku et al., 2011), even though the cytoarchitecture of the avian “cortex” or hyperpallium differs from that of the mammalian neocortex (Medina and Reiner, 2000). Although most of the hyperpallium is considered homologous to the primary visual cortex in mammals (Medina and Reiner, 2000; Reiner et al., 2004; Jarvis et al., 2005), it lacks the neocortical laminar cytoarchitecture consisting of pyramidal cells with apical dendrites spanning multiple layers. Instead it is composed of small, densely packed stellate neurons (Olkowicz et al., 2016) arranged in “pseudo-layers” separated by cell-free laminae, and connected to each other via axonal projections (Medina and Reiner, 2000; Briscoe and Ragsdale, 2018). Although the neuronal cell types in the avian hyperpallium are not organized in a cortical manner, they are homologs to the neurons that make up the mammalian neocortex (Briscoe and Ragsdale, 2018). Consequently, we will refer to the hyperpallium as being cortical hereafter. Starting dorsomedially, the hyperpallium is made up of the hyperpallium apicale (HA), interstitial nucleus of the HA (IHA), hyperpallium intercalatum (HI), and hyperpallium densocellulare (HD) (Atoji et al., 2018). Similar to layer 4 of the neocortex, IHA is the primary recipient of visual input from the dorsal part of the lateral geniculate nucleus [LGN, avian nucleus geniculatus lateralis pars dorsalis (GLd)] (Karten et al., 1973; Watanabe et al., 1983; Wild, 1987; Ng et al., 2010); though HI and, to a lesser extent, HD also receive some input from the GLd. The exact boundary between IHA and HI is poorly defined in the posterior hyperpallium (Karten and Hodos, 1967), and therefore will be referred to collectively as IHA/HI hereafter. Despite the differences in underlying cytoarchitecture, slow-waves during avian NREM sleep show spatio-temporal and propagation properties similar to those found in mammals (Van Der Meij et al., 2019). Moreover, the initiation and propagation of slow-waves primarily occurs in the thalamic input layers of the hyperpallium (i.e., IHA/HI), regions which also show the greatest slow-wave activity (SWA; power in 1.5–4.5 Hz band) during NREM sleep (Van Der Meij et al., 2019), suggesting thalamic involvement in avian cortical slow-waves. Alternatively, neuronal or network properties intrinsic to these regions may account for their greater propensity to initiate slow-waves.

Few studies have examined sleep-related brain activity in birds under anesthesia. The burst suppression pattern has been observed in chickens anesthetized with pentobarbital (Ookawa and Gotoh, 1965; Kadono et al., 1967; Ookawa and Takenaka, 1967; Ookawa, 1972), and zebra finches, chickens, and pigeons anesthetized with isoflurane (Beckers et al., 2014; Mcilhone et al., 2014; Tisdale et al., 2018). However, as no direct comparison of LFP slow-waves occurring during anesthesia and NREM sleep has been conducted across the hyperpallial pseudo-layers, the question remains to what extent slow-waves under anesthesia are comparable to NREM sleep slow-waves. Consequently, we performed intra-cortical recordings covering all pseudo-layers of the visual hyperpallium (including pseudo-layers with and without direct thalamic input) in the same naturally sleeping and isoflurane-anesthetized pigeons.

## Materials and Methods

### Experimental Design

#### Animals

Four adult pigeons (*Columba livia*; two females and two males), implanted for our previous study (Van Der Meij et al., 2019), were used in this study. We chose to work on pigeons, rather than other birds, due to (1) their ability to carry the recording equipment, (2) the availability of a stereotaxic brain atlas (Karten and Hodos, 1967) and extensive neuroanatomical work on this species (Shanahan et al., 2013), and (3) the use of pigeons in earlier work on avian sleep (Van Twyver and Allison, 1972; Walker and Berger, 1972; Tobler and Borbely, 1988; Martinez-Gonzalez et al., 2008). Birds were reared and housed in a breeding aviary. Preceding the start of the electrophysiological procedure, the birds were taken from the colony and housed in pairs in a room with recording aviaries (12 h:12 h light: dark cycle, aviary dimensions: length = 2 m, width = 1 m, height = 2 m). All procedures were performed in accordance with German laws and regulations on animal experiments, and were approved by the Government of Upper Bavaria, according to the Tierschutzgesetz, approval number 55.2-1-54-2532-126-2013.

#### Surgery

Prior to surgery, birds received an injection of diazepam (2 mg/kg) into the breast muscle after which they were anesthetized with isoflurane gas vaporized in oxygen (induction: 3 to 4% and maintenance: 1.5 to 3.5%). Next, the bird's head was fixed in a custom-built stereotaxic frame (i.e., two ear bars and bill clamp) of which the line from the axis of the ear and mouth bar was angled downward 25° relative to the horizontal axis of the stereotaxic frame. The bird's body temperature was maintained around 40°C with a heat pad and checked continuously with a thermometer (Thermalert TH5, Physitemp Instruments Inc., Clifton, NJ) placed underneath the bird's abdomen. To prevent dehydration during the surgery, a subcutaneous injection of saline (0.7–0.9 ml NaCl 0.9% in sterile water) was administered into the neck. Prior to performing a midline incision, head feathers were clipped and Lidocaine gel (2%, as analgesia) was applied to the skin. An initial small window was made in the first layer of the skull to expose the bifurcation point of the mid-sagittal sinus which served as the medial-lateral and anterior-posterior coordinate zero point. In the second skull layer, two small holes were made over the left and right side of the cerebellum for later insertion of the ground and reference wires. A second craniotomy was made on the right side, overlaying the probe insertion site in order to target the posterior visual hyperpallium. Probe implants in the posterior visual hyperpallium (*N* = 4) were placed between 6800 and 7050 μm anterior, and between 1250 and 1360 μm lateral. Due to the use of a smaller size pigeon (Tippler) and different head angle compared to the birds in the pigeon brain atlas (Karten and Hodos, 1967), these coordinates resulted in probe placements in areas histologically comparable with atlas coordinates A10.50 (hyperpallium; including the lower part of HA, IHA/HI, and the top part of HD). An incision was made in the dura for the coronal probe insertion. Preceding probe insertion, multiple small holes (dental acrylic anchor points) were drilled in the first layer of the skull surrounding the insertion side. Additionally the skull surrounding the holes and craniotomies was prepared using Clearfil SE Bond 2 (Kuraray Co., Ltd) for later dental acrylic attachment. The positioning of the probe was completed using a micromanipulator. The precise positioning of the probe depended on avoidance of blood vessels on top of the brain. The probe was slowly lowered, until all 32 channels were inside the brain (i.e., top row of electrode sites approximately 700 μm underneath brain surface). After the probe was in position, the exposed brain and protruding probe shanks were covered using Kwik-Sil (World Precision Instruments). Next, the ground and reference wires were placed between the skull and the dura overlaying the cerebellum. Dental acrylic (Tetric EvoFlow, Ivoclar Vivadent) was used to secure the electrode and connector to the skull. Finally, the skin was sutured around the base of the dental acrylic. In addition, for analgesia, Lidocaine gel was applied to the wound and an intramuscular injection of meloxicam (2 mg/kg) was administered. Finally, the bird received a subcutaneous injection of saline (0.7–0.9 ml).

#### Natural Sleep Recordings

After the pigeon had fully awoken from the surgery, the wireless head stage (Multi Channel Systems, Reutlingen, Germany) was attached and the bird was brought back to the aviary. The birds quickly resumed normal behavior including feeding and flying to and from their perch. Natural sleep recordings were made at least 1 week later and reported in Van Der Meij et al. (2019). In short, neuronal activity was recorded using a 32-channel silicon-based multi-electrode probe (a4 × 8–5 mm-200-400-177, NeuroNexus Technologies, Ann Arbor, MI). The probe consisted of four parallel shanks (shank thickness, 15 μm) separated by 400 μm, each holding 8 recording sites (site surface, 177 μm^2^) spaced 200 μm apart. The resulting matrix of 4 × 8 recording sites thus extended over 1200 × 1400 μm allowing for the simultaneous recordings of HA, IHA/HI, and HD. The probe was referenced to a wire under the skull placed over the cerebellum. The probe's Omnetics connector was attached to a wireless head stage with integrated amplifier (W32, 0.1 Hz−5 kHz bandwidth, 16 bits resolution, 5 kHz sampling rate per channel, 12.5 mV input voltage range, Multi-Channel Systems, Reutlingen, Germany). The head stage was powered by a 900 mAh battery (LiPol Battery Co., Ltd) which was attached to the birds' back with a Velcro strip glued to trimmed feathers. The amplified and digitized signal was sent to a wireless receiver, which in turn was connected to the USB interface board and a data acquisition computer with MC_Rack software (Multi Channel Systems, Reutlingen, Germany). Before the start of each recording session, the battery was placed on the bird's back and attached to the head stage. Neural activity was recorded in natural sleeping pigeons from lights off till lights on (i.e., 12 consecutive hours). The birds were video recorded throughout the night.

#### Isoflurane Anesthesia Recordings

Following the first full night of natural sleep recording (i.e., starting at least 1 week after surgery) the bird was recorded under different levels of isoflurane gas anesthesia according to the following protocol. On the day of recording, birds were food deprived from noon onwards in order to minimize vomiting during and after anesthesia recordings. Then in the evening, birds were taken out of the aviary at lights out (i.e., the same time as the natural sleep recordings would start), equipped with a battery on the back to power the head stage, and were transported to the surgical room. Birds were anesthetized with isoflurane gas vaporized in oxygen (induction: 3–4%, the same professionally calibrated vaporizer was used for all birds). Subsequently, the bird's head was fixed in a custom-built stereotaxic frame (i.e., two ear bars). The bird's body temperature was maintained around 40°C with a heat pad which was only turned on between recordings to minimize electrical noise on the recordings. Peripheral body temperature was checked continuously by a thermometer (Thermalert TH5, Physitemp Instruments Inc., Clifton, NJ) placed underneath the bird's abdomen. All anesthesia recordings were done under dim light and with the experimenter in the surgical room in order to keep an eye on the bird. Starting from the induction level (3 or 4% isoflurane), and going down 0.5% per step, 5 min recordings were made for every isoflurane level. Between each change of isoflurane level, a 5 to 10 min break from recording was taken, during which the heating pad was turned on and time was giving to reach the new anesthesia level. When the minimum level of isoflurane anesthesia required to keep the bird anesthetized (i.e., 1.5%; at lower isoflurane levels, the birds showed signs of arousing such as eye opening and postural changes) was reached and the recording at this level was completed, the isoflurane level was turned back to the induction level for one last high level anesthesia recording. At the end of the recording session, the isoflurane was turned off and the pigeon was allowed to wake up from the anesthesia. After the pigeon had fully awoken, the battery was detached and the bird was returned to the aviary. The bird's behavior was monitored during the next hour to check for complete recovery from the anesthesia. The birds quickly resumed normal behavior including feeding and flying to and from their perch.

#### Anatomy

Prior to implantation, the electrode probes were coated with the fluorescent dye DiI (DiIC 18(3), Invitrogen) for anatomical registration with histological sections. At the end of the study, the brain was removed and frozen for histology to determine probe placement. Frozen brains were cut into 20 μm serial coronal sections using a freezing microtome and mounted on glass slides. Subsequently, DAPI (4′,6-diamidino-2-phenylindole) and Nissl staining was applied. Fluorescence microscopy (Leica) was used to verify probe location in all birds.

### Analysis

#### Signal Filtering

Raw recordings were band-pass filtered (finite impulse response filter) from 1.5 to 200 Hz to yield LFP signals, and high-pass filtered at 350 Hz to yield action potential activity. Based on the analysis in our earlier work (Van Der Meij et al., 2019), a substantial part of the power below 1.5 Hz increased during the first post-operative week and, unlike higher frequency slow-waves (>2 Hz), had an irregular distribution across the recording array. Activity below 1.5 Hz probably reflects an artifact resulting from the development of gliosis around the electrodes (Luan et al., 2017). Hence, to remove this activity, a 1.5 Hz high-pass filter was applied to the signals. As the earlier NREM sleep recordings made before the onset of this artifact showed that hyperpallial slow-wave power peaks around 2 Hz in pigeons (Van Der Meij et al., 2019), the filter applied in this study did not interfere with the slow-wave analysis. Unit activity was only present within the first few days after implantation (Van Der Meij et al., 2019) and could thus not be analyzed in the recordings used in this study, which were made at least one week after surgery.

#### Natural Sleep Scoring

For each bird a full night recording, at least 1 week post-surgery, was analyzed. From this recording, 2 h of “early” night sleep (i.e., 1 h after lights off) and 2 h “late” night sleep (i.e., 2 h before lights on) was extracted. Within these recordings one representative channel, near the center of the array, was chosen for sleep scoring. Raw data of the selected channel was offline low-pass filtered at 100 Hz and down-sampled to 200 Hz using MC_Rack software (Multi Channel Systems, Reutlingen, Germany). Subsequently, sleep states were manually scored by visual inspection of the LFP signal using Somnologica™ (Embla Sleep Diagnostics) and the video recordings, in order to differentiate sleep states and exclude (movement) artifacts. Rather than using scoring epochs, sleep states were scored from the start until the end of each bout of each state. As in previous studies (Martinez-Gonzalez et al., 2008; Lesku et al., 2011), wake was characterized by low amplitude, high frequency LFP signals in combination with waking behaviors; REM sleep by low amplitude, high frequency LFP activity in combination with REM sleep behaviors (e.g., eye closure and head drops); and NREM sleep by immobility accompanied by high amplitude, low frequency (≤4.0 Hz) LFP signals, which had approximately twice the amplitude of alert wakefulness.

#### Anesthesia Scoring

The same electrode site as used for the natural sleep scoring was chosen for the anesthesia scoring. Slow-wave episodes and periods of suppression were manually scored from the start until the end of each bout by visual inspection of the LFP signal using Remlogic™ (Embla Sleep Diagnostics). As not all birds could be recorded at all isoflurane levels, we only analyzed the levels which were present for all birds (i.e., 1.5 to 3.0%).

#### Slow-Wave Analysis

The following analyses were performed on the filtered signals (excluding artifacts and broken electrodes sites) following, with some exceptions, the method previously described in Beckers et al. (2014); Van Der Meij et al. (2019).

Initial visual inspection of the LFP signal was performed by creating waveform plots and spectrograms of each natural sleep and anesthesia recording, in order to directly compare activity during wake, REM, NREM, and isoflurane anesthesia. For visualization, spectrograms were calculated following a multi-taper approach (Thomson, 1982; Prerau et al., 2017), using the “multi_taper_psd” function of the Python (version 3.6) Nitime toolbox (version 0.72) with a bandwidth parameter of 2 Hz and 0.5 s window duration, with time steps of 10 ms.

Spatio-temporal propagation of LFP slow-waves across the 2D-plane of the recording electrode matrix was quantified to examine traveling wave activity (Beckers et al., 2014; Van Der Meij et al., 2019). In short, at 1-ms intervals, LFP waves in the electrode grid that were stronger (i.e., more negative) than the threshold criterion (individual-dependent between −85 and −150 mV) were identified as negative waves, and the changes of their spatial mean in time were tracked. The median number of negative traveling wave “trajectories” that were found in the 2-h NREM sleep recordings was 16609 (range: 14044 to 25691). The median number of negative traveling wave “trajectories” that were found in the lowest anesthesia (1.5% isoflurane; 5 min recording duration) recordings was 648 (range: 502 to 892). To identify and track positive traveling waves the same procedure was followed by selecting LFP waves stronger (i.e., more positive) than the threshold criterion (individual-dependent between 85 and 150 mV).

Videos illustrating the slow-wave patterns were rendered from the band-pass filtered LFP signal (1.5 to 200 Hz) in either real-time or 25x slowed down, in order to capture individual traveling patterns.

#### Spectral Analysis

Power spectral density (PSD) was calculated (using Welch's method; 10.0 Hz bin size) for all episodes of NREM sleep (without wake interruptions) within each 2 h natural sleep (i.e., early and late sleep) recording and for all episodes of anesthesia slow-waves and suppression, separately, within each isoflurane level recording. Then, mean PSD for NREM sleep, and isoflurane bursts and suppression was calculated for each recording site for each bird. From this, the mean integral power in the 1.5–5 Hz (slow-wave activity; SWA), 5–25 Hz and 25–100 Hz (gamma) band was calculated for NREM sleep and slow-wave episodes recorded under the four different isoflurane levels. Given that apparent gliosis around the probes recording sites induced slow artifacts, we were not able to assess frequencies below 1.5 Hz. The relatively large bin size and selected frequency bands in the above mentioned analysis were selected because of the short duration of the slow-wave episodes under the higher isoflurane anesthesia levels, which sets a limit to the length of the window size for spectral analysis.

#### Coherence

Coherence between all possible electrode site pairs was calculated (using Welch's method; 10.0 Hz bin size) for all NREM sleep episodes and slow-wave episodes under anesthesia to examine both global coherence, and coherence between neighboring and distant sites. All coherence calculations were done using the scipy.signal toolbox in Python where coherence is defined as: Cxy = abs(Pxy)^**^2/(Pxx^*^Pyy), with Pxx and Pyy being power spectral density estimates of x and y, and Pxy being the cross spectral density estimate of x and y.

#### Statistics

Statistical analyses were performed in R (version 3.4.4) with the libraries lme4 and lmerTest. Unless noted otherwise, statistical tests were carried out using linear mixed models (LMM), combining early and late NREM sleep, with bird identity as a random factor and isoflurane level as a fixed factor. All numerical values are the mean ± SD, unless otherwise stated.

## Results

We recorded electrical activity from the visual hyperpallium of pigeons, during wake, REM, and NREM sleep, and different levels (1.5, 2.0, 2.5, and 3.0%) of isoflurane anesthesia, using a high-density electrode array connected to a telemetric system. The use of a within-bird design allowed us to compare LFP recordings from the same region in the same bird under natural sleep states and isoflurane anesthesia conditions.

### Comparing Brain Activity During Isoflurane Anesthesia and Natural Wake/Sleep States

**Figure 1:**
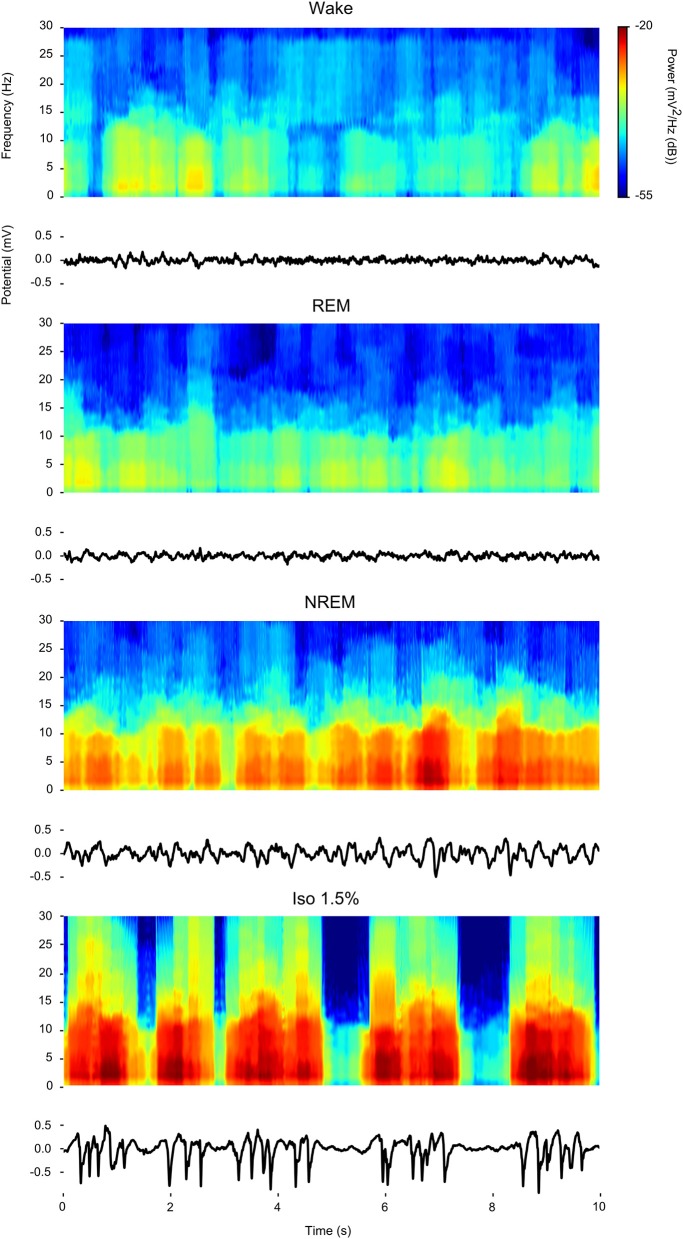
Comparison of power spectral density during wake, rapid eye-movement (REM) sleep, non-rapid eye-movement (NREM) sleep, and low (1.5%) isoflurane anesthesia: For each state a 10 s example waveform **(Bottom)** is plotted with accompanying spectrogram **(Top)**.

Visual inspection of waveforms and accompanying spectrograms of the LFP activity recorded within the same bird during wake, REM, NREM sleep, and isoflurane anesthesia (examples in Figure 1) confirms that LFP activity found under isoflurane anesthesia predominantly consists of slow-waves that appear similar in power distribution to those that are characteristic of NREM sleep and that are much weaker or absent during wake or REM sleep.

### Slow-Wave Characteristics Are Altered Under Isoflurane Anesthesia

**Figure 2:**
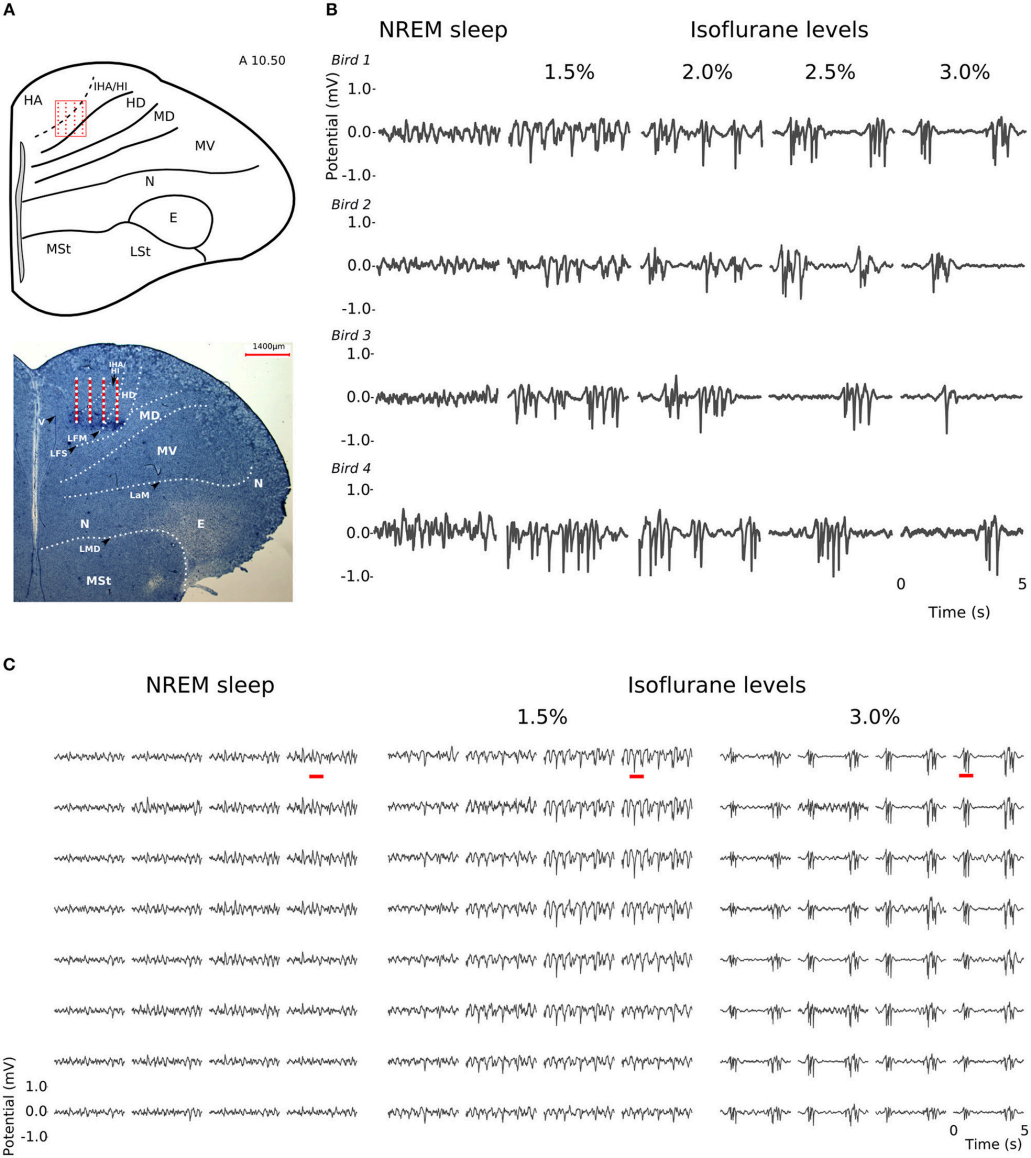
Slow-waves in the hyperpallium during non-rapid eye-movement (NREM) sleep and different levels of isoflurane anesthesia: **(A)** Location of the 32-channel silicon probe. Probes were inserted horizontally in the visual hyperpallium (*N* = 4 birds). The orientation of the electrode grid (red) is depicted with the medial side to the left and the surface of the brain on top. E, entopallium; HA, hyperpallium apicale; HD, hyperpallium densocellulare; HI, hyperpallium intercalatum; IHA, interstitial part of hyperpallium apicale; LaM, lamina mesopallialis; LFM, lamina frontalis suprema; LFS, lamina frontalis superior; LMD, lamina medullaris dorsalis; LSt, striatum laterale; MD/MV, dorsal and ventral mesopallium; MSt, striatum mediale; N, nidopallium. Figure is reproduced from Van Der Meij et al. (2019) by permission of Oxford University Press. **(B)** Five-seconds local field potential (LFP) recording examples from the same electrode site during NREM sleep and the four different levels of isoflurane anesthesia. Each row of waveform plots depicts the recording from a different bird (*Bird 1-4*). Using the same scale for all recording conditions reveals the larger amplitude of the LFP signal during isoflurane recordings. Additionally, LFPs under higher levels of isoflurane show more “spiky” slow-wave episodes interrupted by increasingly longer periods of suppression. **(C)** Five-seconds LFP example recorded from the 4 × 8 electrode array, showing the distribution of oscillations during NREM sleep (left) and under 1.5% (middle) and 3% (right) isoflurane anesthesia. The red underlined episodes in each waveform are shown in more detail in **Figures 7A–C**.

At all levels of isoflurane anesthesia, LFP slow-waves occurred across all recording sites and, as in NREM sleep, had the highest amplitude at the electrode sites corresponding to the thalamo-recipient IHA/HI (based on the stereotaxic coordinates and histology; also see Van Der Meij et al. (2019) (Figures 2A,C). However, under anesthesia, the overall slow-wave waveform had a more “spiky” wave pattern with sharp negative peaks when compared to NREM sleep (Figures 2B,C). Furthermore, waveforms from recordings made under increasing isoflurane levels showed an increase in suppression period duration (LMM; estimate = 0.67337, std. error = 0.09531, *p* = 5.64e-06, duration log-transformed, Supplementary Figure S1A). The duration of suppression periods within each isoflurane recording level was stable. Moreover, there was no difference in suppression duration at 3% isoflurane anesthesia measured at the beginning of the recordings session compared to the end of the recording session (Supplementary Figure S1B).

### Differences in Spectral Composition of Slow-Waves Under Isoflurane Anesthesia

**Figure 3:**
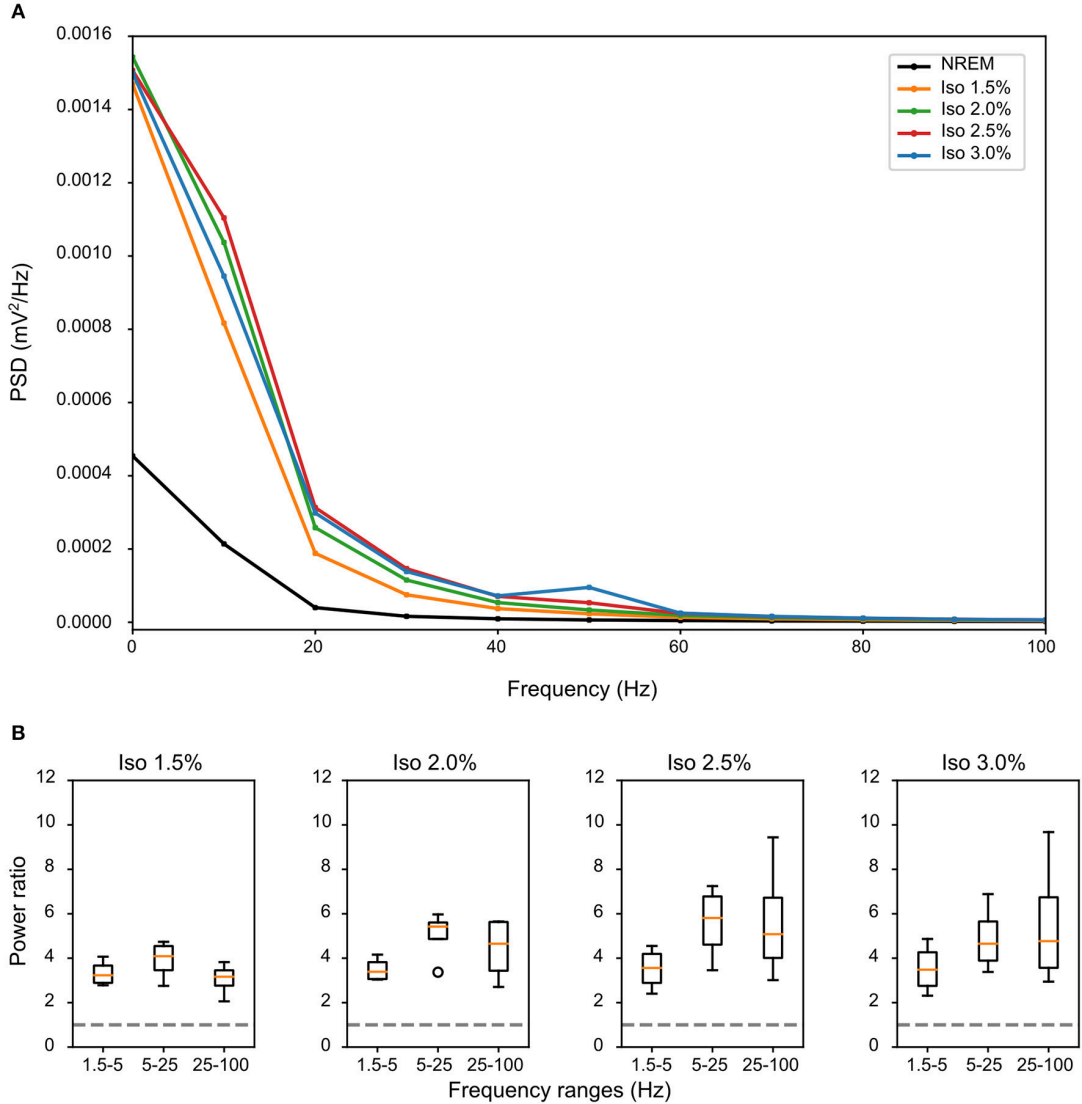
Differences in spectral composition of slow-waves under isoflurane anesthesia: **(A)** Mean power source density (PSD) over all birds and all episodes of non-rapid eye-movement (NREM) sleep (i.e., early and late NREM sleep combined) or all slow-wave episodes under different levels of isoflurane anesthesia. Each condition shows a power frequency distribution typical for pigeon sleep (Martinez-Gonzalez et al., 2008); however, PSD of slow-wave episodes under all levels of isoflurane is higher in all frequency bands when compared to NREM sleep. **(B)** Mean power ratio in the 1.5–5 Hz (slow-wave activity), 5–25 Hz and 25–100 Hz (gamma) bands was calculated over the mean of all slow-wave episodes from all birds (*N* = 4) during NREM sleep and different levels of isoflurane anesthesia. Power in all examined bands was higher during isoflurane anesthesia when compared to NREM sleep (i.e., stipulated line at y-axes = 1). The largest increase in power under increasing levels of isoflurane anesthesia occurred in the 25–100 Hz band.

LFPs recorded in the same bird showed clear slow-waves during both NREM sleep and isoflurane anesthesia, but spectral analysis revealed some differences. When compared to NREM sleep (mean negative amplitude = −0.33 mV, range: −0.08 to −1.04 mV), the amplitude of the negative component of LFPs was significantly lower during anesthesia (mean negative amplitude = −0.57 mV, range: −0.11 to −1.32 mV; log-transformed LMM, estimate = 2.5e−02, std. error = 6.4e−04, *p* < 2e−16, Figures 1, 2B,C). Nonetheless, area specific differences in amplitude observed between IHA/HI and HA/HD during NREM sleep, was sustained under isoflurane anesthesia (Figure 2C). In addition, higher power spectral density (PSD) levels were found in all frequency bins under isoflurane anesthesia (Figure 3A). Specifically, PSD in the slow-wave frequency range (i.e., 1.5–5 Hz) was significantly higher under low (1.5%) isoflurane anesthesia compared to NREM sleep (LMM, estimate = −1.0e−3, std. error = 1.1e−4, *p* = 7.5e−4). As some of the waveform plots depicting suppression states showed a low amplitude LFP signal, a PSD analysis was also performed on the suppression states. PSD in all frequency bands was lower than during NREM sleep. However, there was still considerably more power in the 1.5–5 Hz range than in the higher frequency ranges.

**Figure 4:**
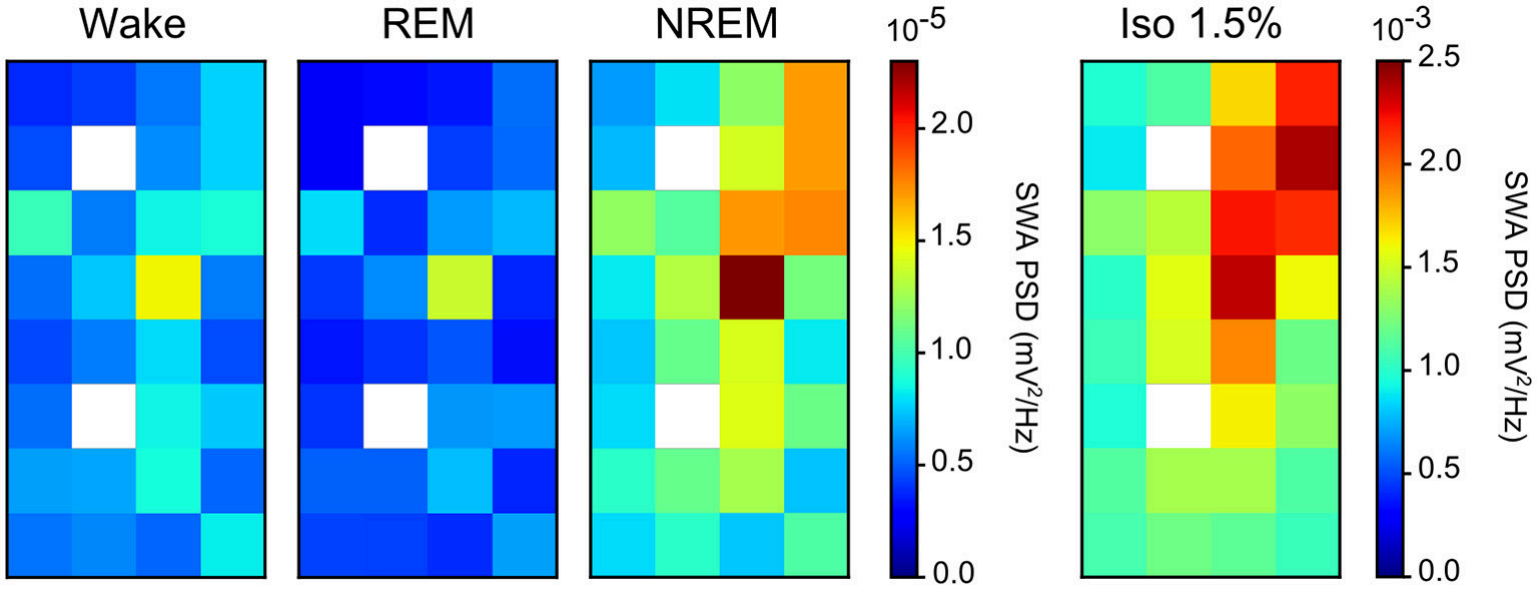
Slow-wave activity (SWA) during both non-rapid eye-movement (NREM) sleep and isoflurane anesthesia: Mean SWA (1.5–5 Hz power; *N* = 4 birds) over all episodes of wake, rapid eye-movement (REM) sleep, NREM sleep, and low (1.5%) isoflurane episodes. SWA during NREM sleep and low anesthesia is the highest in the diagonal of the recording array (i.e., on the electrode sites that were placed in IHA/HI, the thalamic input layer). Image plots of SWA activity under 2.0, 2.5, and 3.0% isoflurane anesthesia showed the same pattern as the 1.5% anesthesia image plot (data not shown).

Comparing the mean power ratio in the frequency bands 1.5–5 Hz (SWA; slow-wave activity), 5–25 Hz and 25–100 Hz (gamma) over all slow-wave periods from all birds (*N* = 4) during NREM sleep and different levels of anesthesia, we found that the power was higher in all examined bands during isoflurane anesthesia compared to NREM sleep. In addition, comparing the power ratio in each examined frequency range under increasing levels of isoflurane revealed that the largest increase in power under anesthesia occurred in the 5–25 Hz and 25–100 Hz band (Figure 3B). The power in the slow-wave activity range (i.e., 1.5–5 Hz) did not change with increasing anesthesia levels (Figure 3B). Looking at the spatial distribution of SWA power over the electrode grid, we found that the same electrode sites (i.e., the ones placed in IHA/HI) that showed the highest SWA during NREM sleep also showed the highest SWA under isoflurane anesthesia (Figure 4).

### Slow-Wave Coherence Increased Under Isoflurane Anesthesia

**Figure 5:**
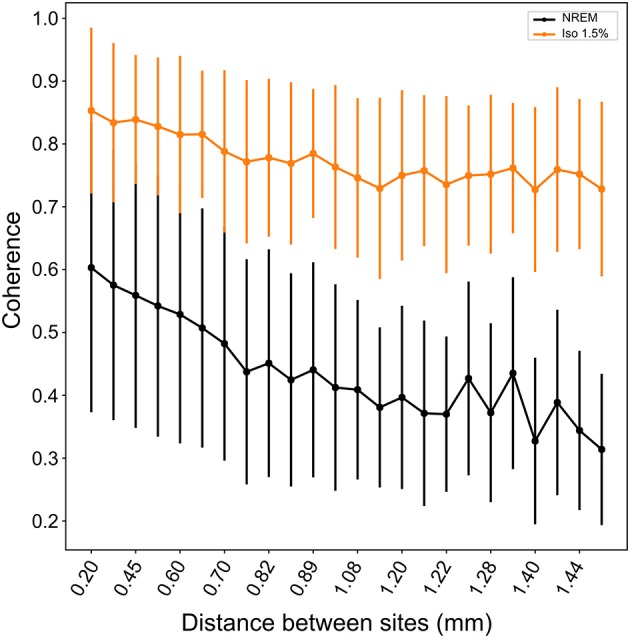
Coherence between neighboring and distance sites during NREM sleep and isoflurane anesthesia: Mean coherence (dots; whiskers are SD) in the 1.5–5Hz frequency band decreases with increasing distance between electrode sites. Nonetheless, coherence is significantly higher under all levels of isoflurane anesthesia (1.5% isoflurane shown here; comparable results for higher isoflurane levels) compared to NREM sleep.

Comparing the waveforms of LFP slow-waves under NREM sleep and different anesthesia levels suggested that slow-waves appear to be more spatially synchronized between electrode recording sites during anesthesia. To quantify this, we calculated the mean coherence in the 1.5–5Hz frequency band between sites over all LFP slow-wave events per NREM sleep and low (1.5%) anesthesia recordings. Mean coherence between sites during NREM sleep is 0.49 (range: 0.39 to 0.65) while during low anesthesia it is 0.8 (range: 0.67 to 0.85). We additionally examined coherence between electrode sites as a function of distance between sites and state (i.e., NREM sleep and anesthesia levels). Coherence was significantly higher under all anesthesia levels when compared to NREM sleep (LMM; estimate = 0.21578, std. error = 0.05694, *p* = 0.0289, Figure 5), and coherence overall decreased as distance between sites increased (slope = −0.127883, *p* ≤ 2e−16).

### Comparable Slow-Wave Propagation Under Isoflurane Anesthesia

**Figure 6:**
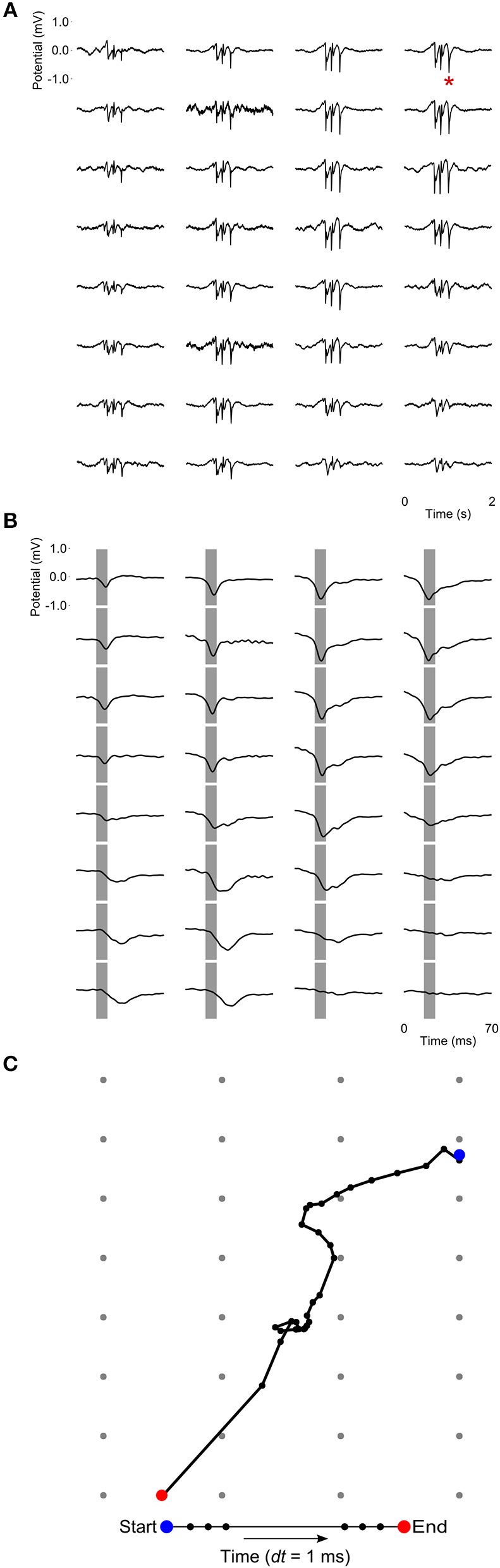
Propagating slow-waves during anesthesia: **(A)** A 2 s waveform example recorded during high (3.0%) isoflurane anesthesia, showing that oscillations appear to be globally distributed across the recording array. **(B)** Detail of the LFP peak indicated with a red asterisk in **(A)**, showing that peak activity actually occurs at slightly different times in different sites, especially along the diagonal of the recording plane, corresponding to IHA/HI. Gray bars depict the negative component of the wave and are centered on the negative peak of the site indicated with a red asterisk in **(A)**. **(C)** LFP peak propagation across the grid is tracked by calculating the center of gravity of the propagating slow-wave in 1-ms intervals, based on sites with a potential < −0.15 mV.

**Figure 7:**
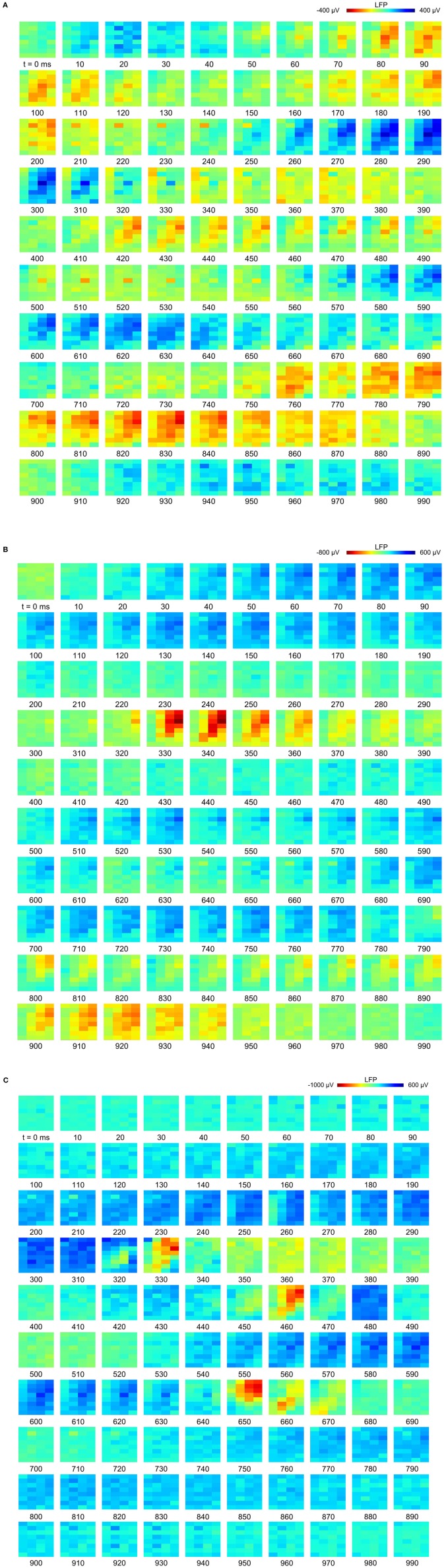
Propagation of slow-waves during non-rapid eye-movement (NREM) sleep and isoflurane anesthesia: The red underlined episodes in Figure 2C are visualized in a sequence of image plots where pixels represent electrode sites and electrical potential is coded in color. Slow-wave propagation patterns during **(A)** NREM sleep and, **(B,C)** 1.5 and 3.0% isoflurane anesthesia. LFP (local field potential) activity generally initiates along the diagonal of the recording plane, corresponding to IHA/HI, and propagates mostly within the diagonal under all recording conditions. LFP voltage scale differs across subfigures **(A–C)**.

**Figure 8:**
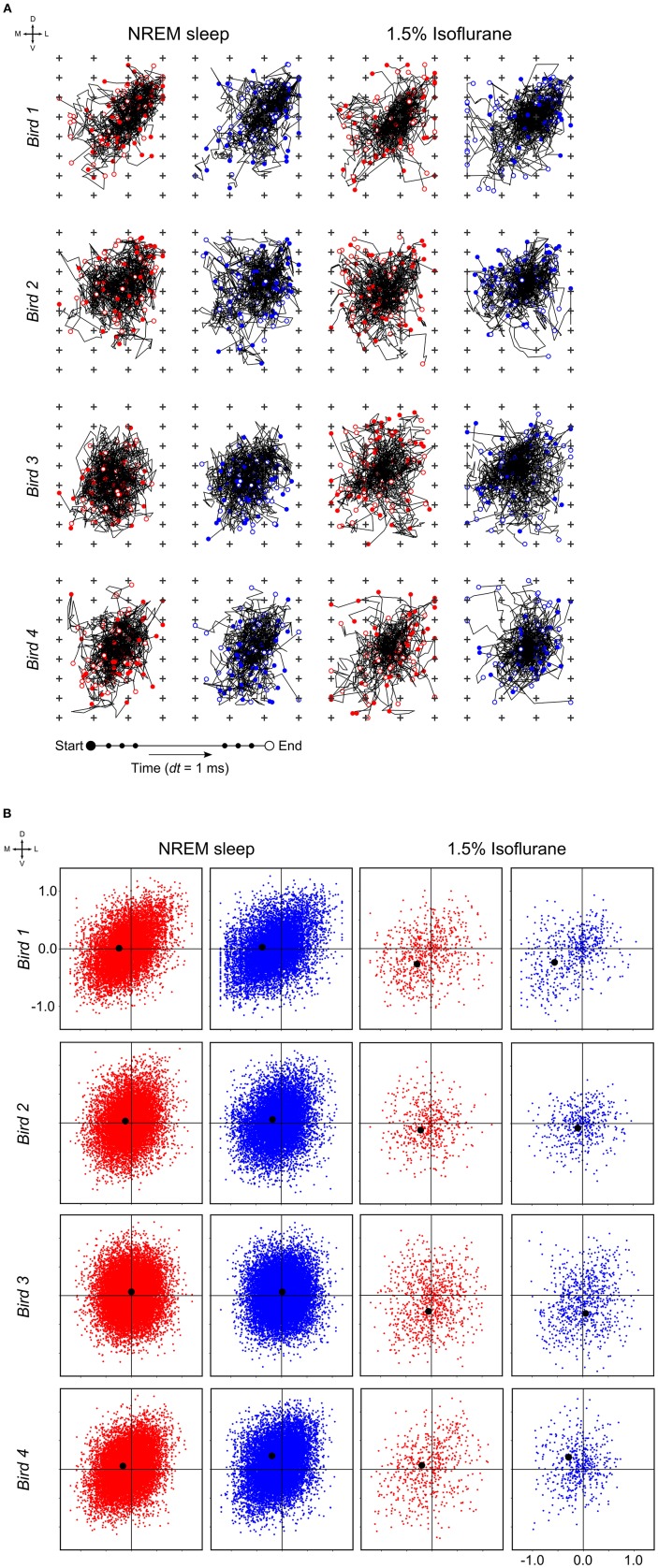
Slow-wave trajectories and propagation direction: **(A)** Wave trajectories along the 2D-plane of the recording array (*N* = 50 random waves; plus signs depict electrode sites) during non-rapid eye-movement (NREM) sleep (the early night results shown here and comparable to late night results) and under 1.5% isoflurane anesthesia. **(B)** Net wave propagation (in mm) was calculated for every local field potential (LFP) slow-wave in a 2 h recording of NREM sleep (the early night results shown here and comparable to the late night results) and 5 min 1.5% isoflurane anesthesia recording from the same bird. Shown are negative (red dots), positive waves (blue dots) and mean propagation direction (black dot). Each row of graphs depicts the results of a different bird (*N* = 4).

At a time scale of seconds, during both NREM sleep and anesthesia recordings, the LFP waveforms appear the same across the array (Figure 6A), almost as if the oscillations occur near-synchronously across the different “pseudo-layers.” However, at the time scale of tens of milliseconds, and zooming in on a single LFP peak from Figure 6A, shows that this peak occurs at slightly different times across the electrode grid (Figure 6B) with the activity peak shifting later along the diagonal of the array. In general, like NREM sleep, LFP slow-waves under isoflurane anesthesia primarily propagate along the IHA/HI, with occasional propagation to the overlying HA and, to a lesser extent, the underlying HD. This is also evident when slow-wave LFPs across the electrode matrix are plotted in a time series of image plots (Figures 7A–C, Supplementary Figure S2 and Supplementary Videos 1–3). Moreover, tracing the trajectories of the center of gravity of propagating slow-waves shows that slow-waves under anesthesia, as during NREM sleep, often occur first along the diagonal of the recording plane, corresponding to IHA/HI (Figures 6C, 8A and Supplementary Figures 3A, B). When the net movement of each slow-wave across the plane of the electrode grid was expressed as a mean vector, the group mean vectors for both positive and negative component of LFP slow-waves showed significantly non-random directions (Rayleigh-tests; *p* < 0.001) in every recording (*N* = 8, 2 h natural sleep recordings and *N* = 16, 5 min anesthesia recordings at four different levels from 4 birds). The mean group vectors under low (1.5%) isoflurane anesthesia show small, non-significant differences in direction (paired *t*-tests: df = 3, *p* > 0.1) from the mean group vectors under NREM sleep (black dots in Figure 8B). Nonetheless, mean vector length for positive slow-waves under both NREM sleep (0.39 ± 0.25 mm) and low (1.5%) isoflurane anesthesia (0.48 ± 0.28 mm) is not significantly different (paired *t*-test: df = 3, *p* = 0.09) and mean vector length for negative waves under NREM sleep (0.36 ± 0.23 mm) and low (1.5%) isoflurane anesthesia (0.53 ± 0.28 mm) showed a slight, but significant difference (paired *t*-test: df = 3, *p* = 0.01) while there remains large variation in the direction of individual slow-waves (Figure 8B).

**Figure 9:**
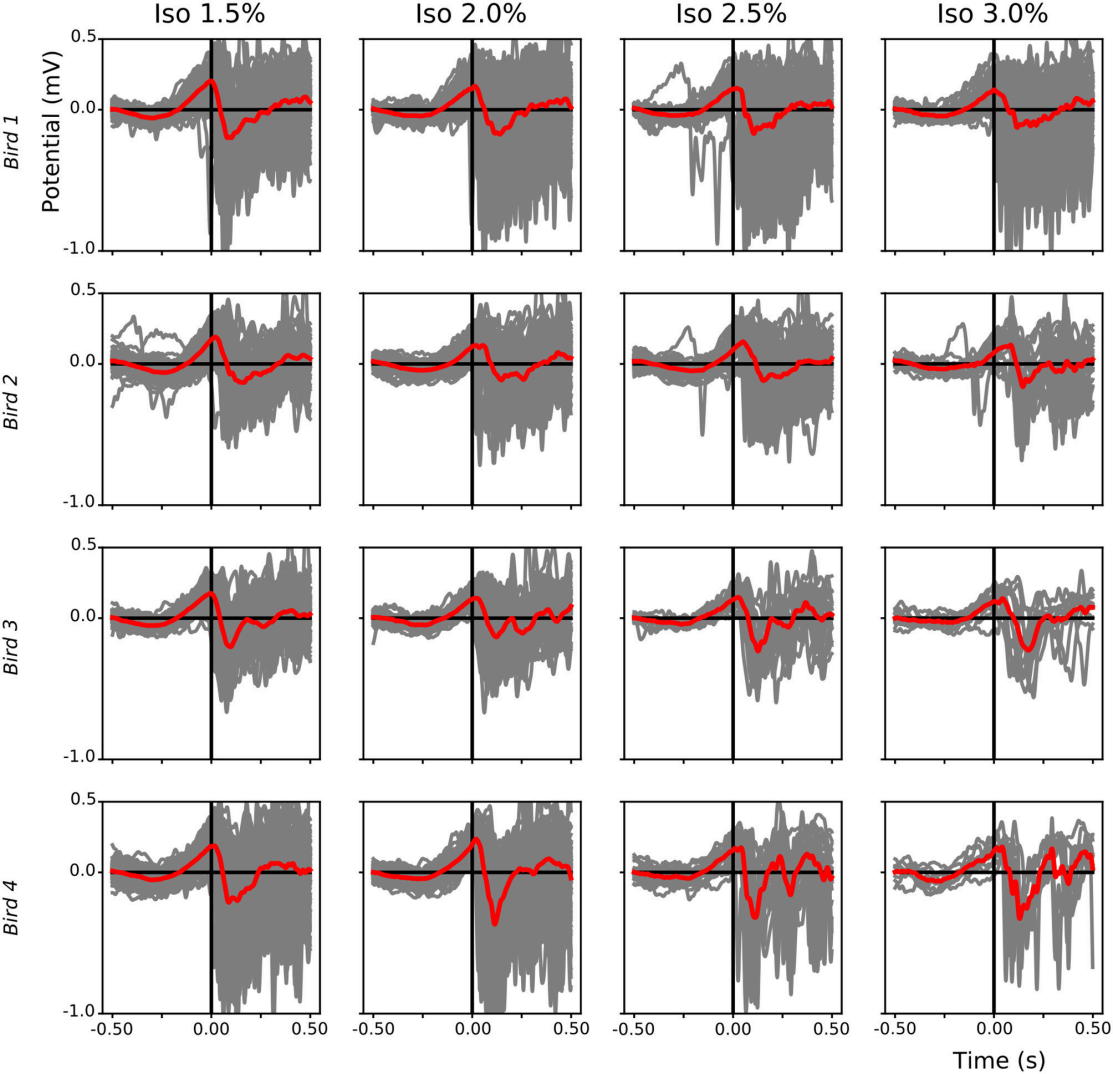
Leading positive component of local field potential (LFP) slow-waves during isoflurane anesthesia: For each bird (different rows) the start of each slow-wave episode after a suppression period during different isoflurane levels was plotted (gray lines, mean in red). Slow-waves after a period of suppression start with a positive component followed by a sharp negative component.

The alternation of slow-waves and suppression periods under isoflurane anesthesia, compared to the almost continuous slow-wave pattern during NREM sleep, allowed us to investigate which component (i.e., negative or positive) of the LFP leads during slow-wave propagation under anesthesia. Superimposition of waveform plots of the first slow-wave after a suppression period (alignment based on scored start time of each single slow-wave period) showed that, during low (1.5%) anesthesia, a positive component of the field leads with respect to the negative component (Figure 9).

## Discussion

Our understanding of the similarities and differences between slow-waves occurring under different types of anesthesia and NREM sleep has been primarily based on EEG, LFP, and intracellular recordings in cats (Steriade et al., 1993a,b,c; Chauvette et al., 2011). Based on those studies, anesthesia is now a well-established method for studying the cortical dynamics of slow-waves when experiments in freely behaving animals are technically impossible. Given that birds show similar sleep states to mammals, anesthesia is also used in avian sleep research (Beckers et al., 2014). Moreover, anesthesia (often isoflurane) is used in other fields, such as the neuroscience of birdsong (e.g., Beckers and Gahr, 2012; Menardy et al., 2014). Although there is EEG evidence for parallels between NREM sleep and isoflurane anesthesia in birds (Tisdale et al., 2018), a comparison of the underlying intra-cortical brain activity has been lacking. Using intra-cortical high-density electrode array recordings from the same naturally sleeping and isoflurane anesthetized pigeons, we characterized and compared the spatio-temporal properties of slow-waves in the avian hyperpallium occurring during both states.

### Effects of Isoflurane Anesthesia on Slow-Waves

Our intra-cortical recordings of pigeons revealed that slow-waves occur at all isoflurane anesthesia levels examined, although the duration of slow-wave episodes becomes shorter and the periods of suppression between them become longer, as the level increases. Slow-waves under isoflurane anesthesia are more similar in spatial power distribution to NREM sleep than to wake or REM sleep. In addition, as in NREM sleep, slow-waves under anesthesia propagate through the hyperpallium following the same spatio-temporal patterns including primary slow-wave initiation and propagation in the thalamic input layer IHA/HI. Interestingly, the analysis of the first slow-wave after isoflurane induced periods of suppression revealed that slow-waves under anesthesia have a leading positive component followed by a negative component. Although this might reflect a dipole passing the 2D-plane of the array, three dimensional recordings during NREM sleep and intra-cellular recordings are needed to fully understand the spatial distribution of positive and negative fields.

Despite the similarities between slow-waves occurring during NREM sleep and isoflurane anesthesia, a number of differences were observed. First, under anesthesia, periods of suppression emerged in between slow-waves. The time spent in suppression increased with increasing anesthesia levels, a finding that has also previously been reported in rats and chickens anesthetized with isoflurane (Murrell et al., 2008; Mcilhone et al., 2014). Consequently, brain activity during the lowest level of isoflurane (i.e., 1.5%) possible to measure from in this study most closely resembles NREM sleep. The low amplitude activity visible during some episodes of suppression states are of unknown origin but might be caused by local processes, artifacts on the recordings sites or volume conduction from multiple, dispersed sources further away in the same hemisphere or from the left hemisphere, as slow-waves (i.e., burst state) can occur asynchronously between hemispheres (Rattenborg and Amlaner, 2010; Tisdale et al., 2018). Second, the LFP negative peak amplitude was significantly larger (i.e., more negative) during anesthesia when compared to NREM sleep. Third, PSD significantly increased in all examined frequency bands (1.5–100 Hz) under isoflurane anesthesia, with the highest increase taking place in the 5–25 and 25–100 Hz bands. Nonetheless, the power spatial distribution in the slow-wave frequency band (i.e., 1.5–5 Hz) under low (1.5%) isoflurane anesthesia is more comparable to NREM sleep than to wake and REM sleep, suggesting that slow-waves during isoflurane anesthesia are similar, though stronger, than slow-waves under NREM sleep. This finding is in agreement with a recent EEG study in pigeons (Tisdale et al., 2018). Interestingly, cats anesthetized with ketamine-xylazine exhibit lower power in the low frequencies (0.1–4 Hz and 8–14 Hz) when compared to NREM sleep (Chauvette et al., 2011). Whether this difference in power spectra is due to the use of different anesthetics, or to the species examined, will need to be determined in future studies. Lastly, slow-wave coherence between electrode sites was higher under all levels of anesthesia when compared to NREM sleep. Interestingly, higher coherence in the slow-wave frequency range during anesthesia compared to NREM sleep was also found in cats anesthetized with ketamine-xylazine (Chauvette et al., 2011). Additional studies will have to determine whether increased coherence in the slow-wave frequency range is a common feature of anesthetics.

### Conclusion, Implications, and Future Perspectives

To our knowledge, this is the first direct comparison of intra-cortical brain activity during wake, NREM sleep, and REM sleep, and different levels of isoflurane anesthesia in birds. We opted for isoflurane in this study as this is a common anesthetic used in lab studies examining the neurophysiology of avian sleep (e.g., Beckers et al., 2014, Tisdale et al., 2018) and the neurological correlates of bird song (e.g., Beckers and Gahr, 2012; Menardy et al., 2014). Nonetheless, future research should consider examining other anesthetics (Zhang et al., 2015). Furthermore, as read-outs of vaporizer output levels of anesthesia could be imprecise, future experiments should monitor the blood level of the anesthetic. Nonetheless, this initial study demonstrates that, as in a number of mammals, slow-waves in pigeons anesthetized with isoflurane are similar in some respects to those occurring during NREM sleep. In particular, the primary propagation pattern of LFP slow-waves along the IHA/HI, a layer receiving thalamic input, under isoflurane anesthesia and during NREM sleep could indicate thalamic involvement in the genesis of cortical slow-waves during both states. Nonetheless, periods of suppression, higher coherence, and increased high-frequency activity, clearly distinguished isoflurane anesthesia from NREM sleep. These considerable differences might be important to take into consideration when attempting to interpret data from studies utilizing anesthesia in neurophysiological experiments.

## Data Availability

The datasets generated for this study are available on request to the corresponding author.

## Author Contributions

JvdM, GB, and NR contributed to conception and design of the study. JvdM and DM-G collected the data. JvdM, DM-G, GB, and NR analyzed data. JvdM and GB performed the statistical analyses. JvdM wrote the first draft of the manuscript. All authors contributed to manuscript revision, read, and approved the submitted version.

### Conflict of Interest Statement

The authors declare that the research was conducted in the absence of any commercial or financial relationships that could be construed as a potential conflict of interest.

## Supplementary Material

Supplementary Figure S1 Progression of suppression durations at the different isoflurane levels: **(A)** Mean suppression duration (black dot, standard error bars in grey) becomes longer with increasing anesthesia level. **(B)** Normalized suppression duration per isoflurane level (colored circles) and per bird (different graphs). Duration of suppression episodes was stable within each isoflurane recording level. Moreover, there was no difference between suppression duration at 3% isoflurane anesthesia measured at the beginning of the recording session (blue circles) compared to the ending of the recording session (black circles; i.e., turning back to 3% after recording at 1.5%). Note that differences in circles plotted along the x-axis are due to slight variation in recordings duration per level and bird.

Supplementary Figure S2 High temporal resolution (2 ms) plots of propagating slow-waves during low (1.5%) and high (3.0%) isoflurane anesthesia depicted in Figure 7B (frame 320–380) and Figure 7C (frame 650–670), respectively. Similar to NREM sleep, the local field potential (LFP) activity under anesthesia generally initiates along the diagonal of the recording plane, corresponding to IHA/HI, and propagates mostly within this diagonal.

Supplementary Figure S3 Slow-wave trajectories and propagation direction during higher isoflurane levels: **(A)** Wave trajectories along the 2D-plane of the recording array (*N* = 50 or less random waves; plus signs depict electrode sites) under 2.0, 2.5, and 3.0% isoflurane anesthesia, which are comparable to the trajectory plots depicted in Figure 8A. **(B)** Net wave propagation (in mm) was calculated for every local field potential (LFP) slow-wave in a five minute isoflurane anesthesia recording (2.0, 2.5, and 3.0%, respectively) from the same birds as depicted in Figure 8B. Shown are negative (red dots), positive waves (blue dots) and mean propagation direction (black dot). Each row of graphs depicts the results of a different bird (*N* = 4).

Supplementary Video S1 Video (slowed down 25x) of the image sequence shown in Figure 7A. Slow-wave propagation pattern during natural NREM sleep.

Supplementary Video S2 Video (slowed down 25x) of the image sequence shown in Figure 7B. Slow-wave propagation pattern under 1.5% isoflurane anesthesia.

Supplementary Video S3 Video (slowed down 25x) of the image sequence shown in Figure 7C. Slow-wave propagation pattern under 3.0% isoflurane anesthesia.
