# Supplementary material for: Neurophysiology of Avian Sleep: Comparing Natural Sleep and Isoflurane Anesthesia
Source: Front Neurosci. 2019 Mar 28;13:262. doi: 10.3389/fnins.2019.00262 (PMC6447711; doi:10.3389/fnins.2019.00262)
Supplement: Supplementary Figure S1 — Progression of suppression durations at the different isoflurane levels: (A) Mean suppression duration (black dot, standard error bars in grey) becomes longer with increasing anesthesia level. (B) Normalized suppression duration per isoflurane level (colored circles) and per bird (different graphs). Duration of suppression episodes was stable within each isoflurane recording level. Moreover, there was no difference between suppression duration at 3% isoflurane anesthesia measured at the beginning of the recording session (blue circles) compared to the ending of the recording session (black circles; i.e., turning back to 3% after recording at 1.5%). Note that differences in circles plotted along the x-axis are due to slight variation in recordings duration per level and bird. [file Data_Sheet_1.PDF]

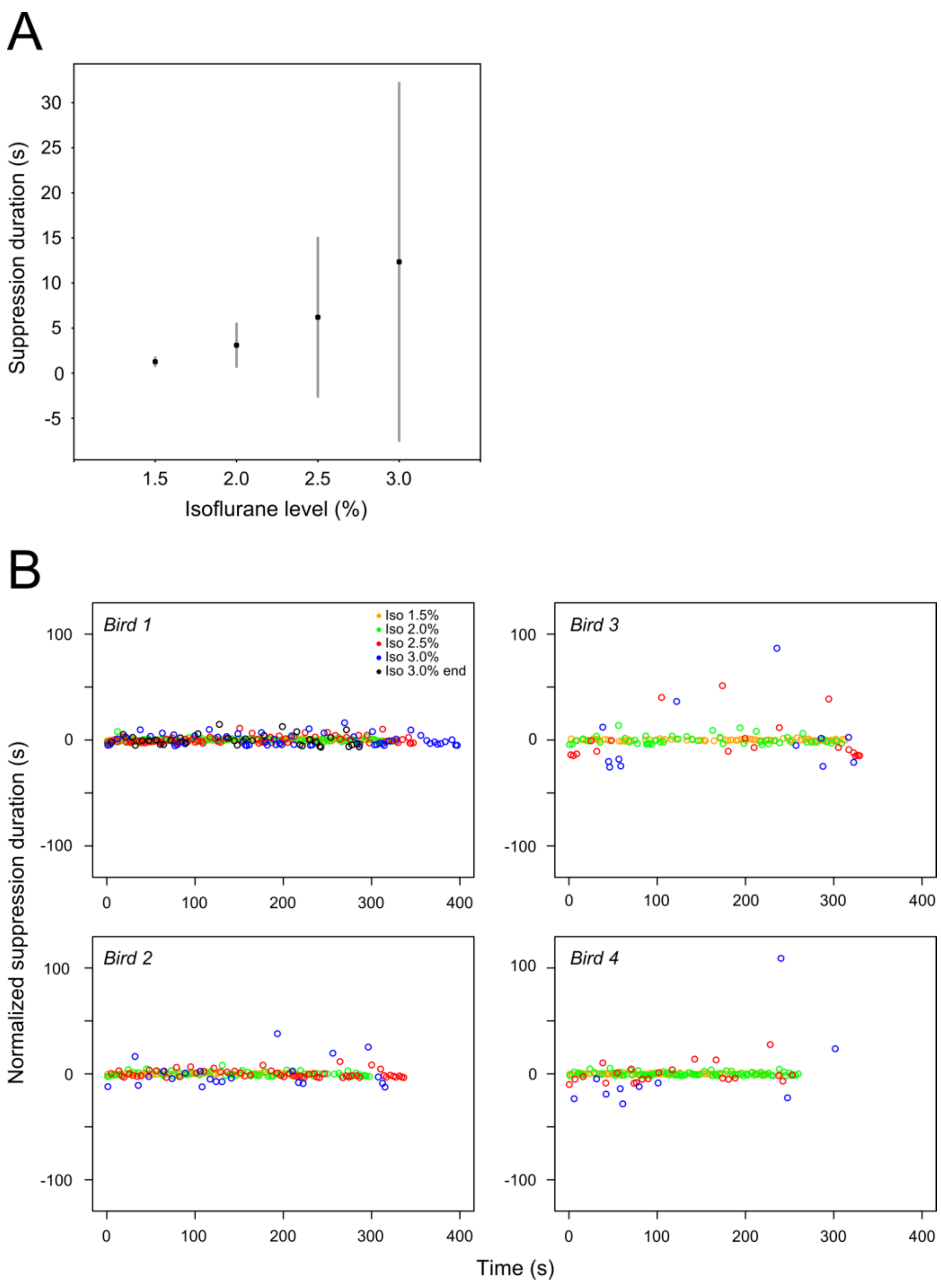

Supplementary Figure S1

Progression of suppression durations at the different isoflurane levels: **(A)** Mean suppression duration (black dot, standard error bars in grey) becomes longer with increasing anesthesia level. **(B)** Normalized suppression duration per isoflurane level (colored circles) and per bird (different graphs). Duration of suppression episodes was stable within each isoflurane recording level. Moreover, there was no difference between suppression duration at 3% isoflurane anesthesia measured at the beginning of the recording session (blue circles) compared to the ending of the recording session (black circles; i.e. turning back to 3% after recording at 1.5%). Note that differences in circles plotted along the x-axis are due to slight variation in recordings duration per level and bird.
