# Supplementary material for: Neurophysiology of Avian Sleep: Comparing Natural Sleep and Isoflurane Anesthesia
Source: Front Neurosci. 2019 Mar 28;13:262. doi: 10.3389/fnins.2019.00262 (PMC6447711; doi:10.3389/fnins.2019.00262)
Supplement: Supplementary Figure S2 — High temporal resolution (2 ms) plots of propagating slow-waves during low (1.5%) and high (3.0%) isoflurane anesthesia depicted in Figure 7B (frame 320–380) and Figure 7C (frame 650–670), respectively. Similar to NREM sleep, the local field potential (LFP) activity under anesthesia generally initiates along the diagonal of the recording plane, corresponding to IHA/HI, and propagates mostly within this diagonal. [file Data_Sheet_2.PDF]

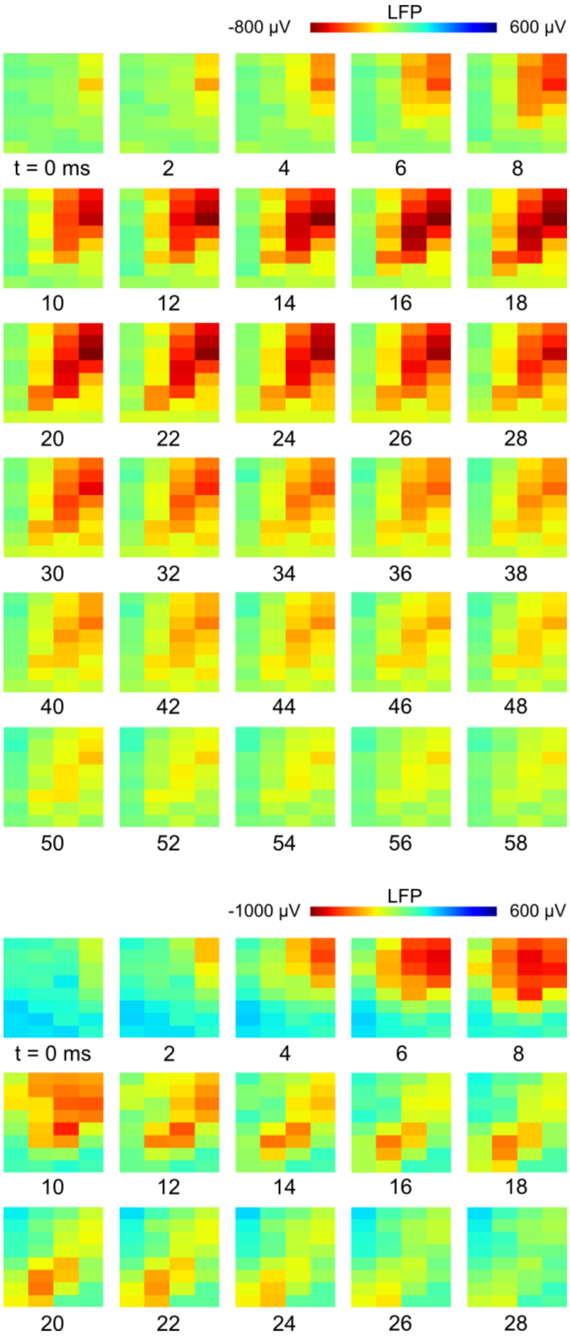

Supplementary Figure S2

High temporal resolution (2 ms) plots of propagating slow-waves during low (1.5%) and high (3.0%) isoflurane anesthesia depicted in figure 7B (frame 320-380) and 7C (frame 650-670), respectively. Similar to NREM sleep, the local field potential (LFP) activity under anesthesia generally initiates along the diagonal of the recording plane, corresponding to IHA/HI, and propagates mostly within this diagonal.
