# Supplementary material for: Neurophysiology of Avian Sleep: Comparing Natural Sleep and Isoflurane Anesthesia
Source: Front Neurosci. 2019 Mar 28;13:262. doi: 10.3389/fnins.2019.00262 (PMC6447711; doi:10.3389/fnins.2019.00262)
Supplement: Supplementary Figure S3 — Slow-wave trajectories and propagation direction during higher isoflurane levels: (A) Wave trajectories along the 2D-plane of the recording array (N = 50 or less random waves; plus signs depict electrode sites) under 2.0, 2.5, and 3.0% isoflurane anesthesia, which are comparable to the trajectory plots depicted in Figure 8A. (B) Net wave propagation (in mm) was calculated for every local field potential (LFP) slow-wave in a five minute isoflurane anesthesia recording (2.0, 2.5, and 3.0%, respectively) from the same birds as depicted in Figure 8B. Shown are negative (red dots), positive waves (blue dots) and mean propagation direction (black dot). Each row of graphs depicts the results of a different bird (N = 4). [file Data_Sheet_3.PDF]

A

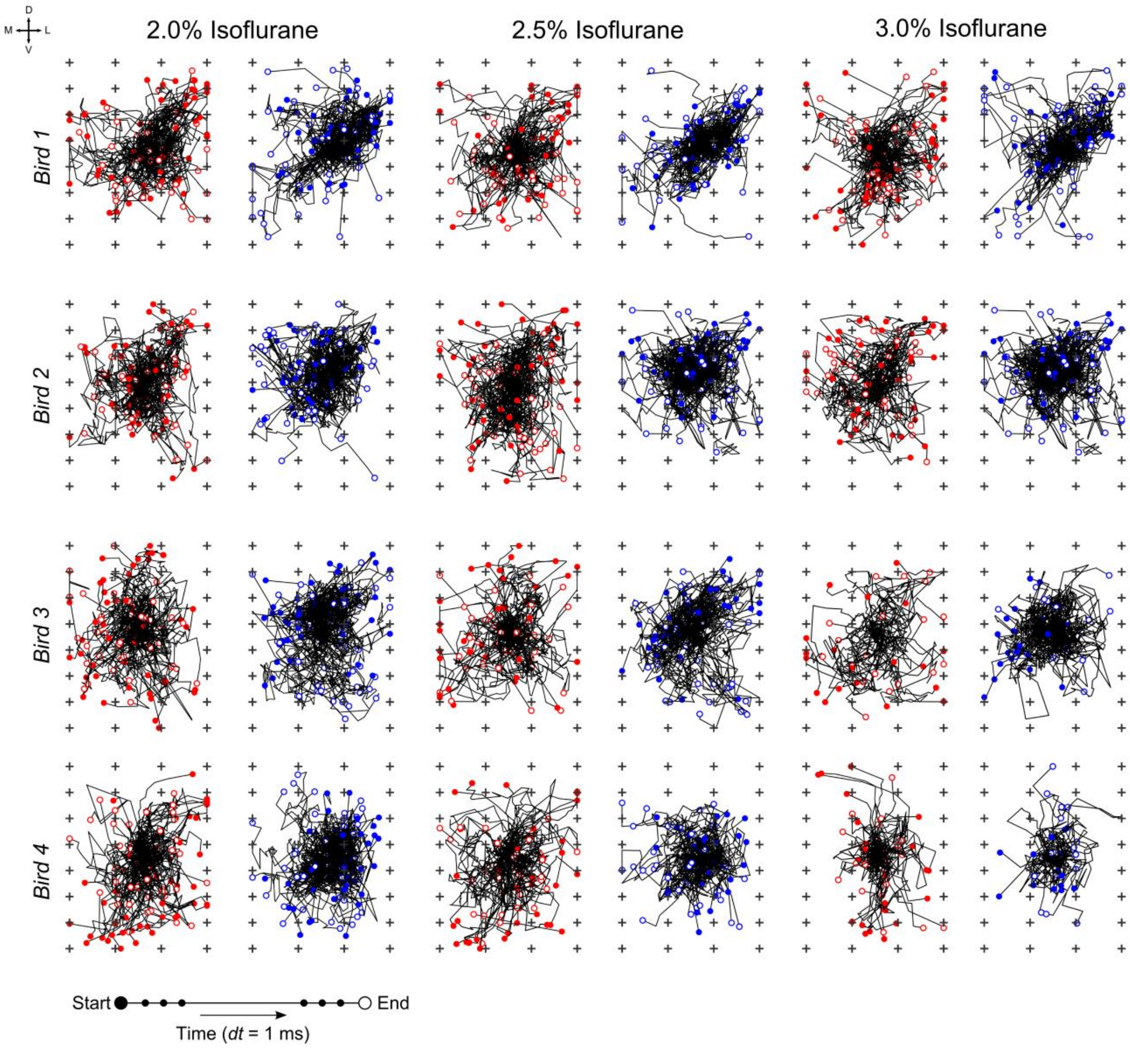
D
M
L
V
2.0% Isoflurane
2.5% Isoflurane
3.0% Isoflurane
Bird 1
Bird 2
Bird 3
Bird 4
Start
End
Time (dt = 1 ms)

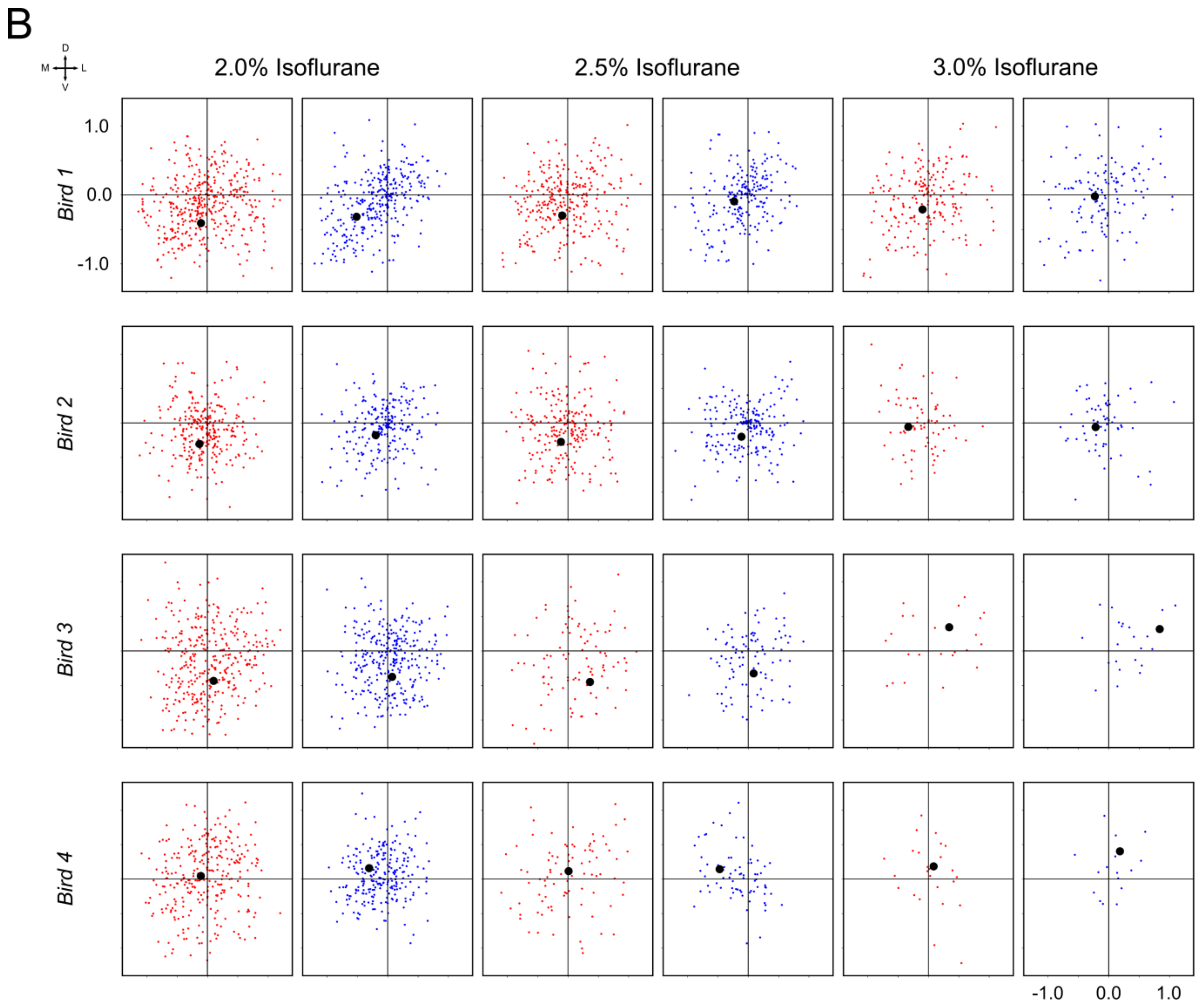

Supplementary Figure S3

Slow-wave trajectories and propagation direction during higher isoflurane levels: **(A)** Wave trajectories along the 2D-plane of the recording array (N=50 or less random waves; plus signs depict electrode sites) under 2.0%, 2.5% and 3.0% isoflurane anesthesia, which are comparable to the trajectory plots depicted in Figure 8A. **(B)** Net wave propagation (in mm) was calculated for every local field potential (LFP) slow-wave in a five minute isoflurane anesthesia recording (2.0%, 2.5% and 3.0%, respectively) from the same birds as depicted in Figure 8B. Shown are negative (red dots), positive waves (blue dots) and mean propagation direction (black dot). Each row of graphs depicts the results of a different bird (N= 4).
